# Preoperative morphological analysis by transesophageal echocardiography and predictive value of plasma landiolol concentration during systolic anterior motion mitral valve repair : a report of three cases

**DOI:** 10.1007/s00540-013-1731-4

**Published:** 2013-10-27

**Authors:** Manabu Yoshimura, Takayuki Kunisawa, Takafumi Iida, Megumi Matsumoto, Hayato Takakai, Hirotsugu Kanda, Atsushi Kurosawa, Osamu Takahata, Hiroshi Iwasaki

**Affiliations:** Department of Anesthesiology and Critical Care Medicine, Asahikawa Medical University, Midorigaoka-higashi 2-1-1-1, Asahikawa, Hokkaido 078-8510 Japan

**Keywords:** Systolic anterior motion of the mitral valve (SAM), Transesophageal echocardiography (TEE), Mitral valve annuloplasty (MVP), Landiolol hydrochloride

## Abstract

We report three cases with systolic anterior motion (SAM) after mitral valve plasty. Preoperative mitral valve morphology is a risk factor for SAM. The morphological characteristics of SAM have been revealed in several studies. We found a small distance between coaptation and the interventricular septum in all cases, and cases 2, and 3 had a low AL/PL ratio, whereas case 3 had a large PML, which was revealed by transesophageal echocardiography. With the use of 3D transesophageal echocardiography, when mitral valve prolapse was investigated, in all three cases, it was easy to specify lesions. The issue for the future is 3D analysis when SAM is occurring.

## Introduction

Systolic anterior motion (SAM) of the mitral valve has been described after mitral repair in patients with mitral regurgitation (MR) [1, 2]. The occurrence of SAM leads to left ventricular outflow obstruction (LVOTO), mitral regurgitation, and severe hemodynamic instability. Several studies have revealed the morphological characteristics of SAM by intraoperative transesophageal echocardiography (TEE) [3] [4] [5].

Landiolol is an ultra-short-acting β1 selective adrenoceptor antagonist, with a short plasma half-life of 4 min [6], and decreases heart rate during cardiac surgery. The landiolol concentration reaches a rapid steady state level, and rapidly decreases after complete administration [7]. Therefore, it has been recommended for treating SAM [8]. A population pharmacokinetic model of landiolol has been developed in healthy subjects [9]. Using those parameters, we obtained plasma landiolol concentrations during perioperative anesthetic management using the Stanpump software.

We here describe three patients with SAM who were treated with landiolol, and analyzed SAM morphological characteristics by TEE and predicted landiolol plasma concentration with the disappearance of SAM.

## Case 1

**Fig. 1:**
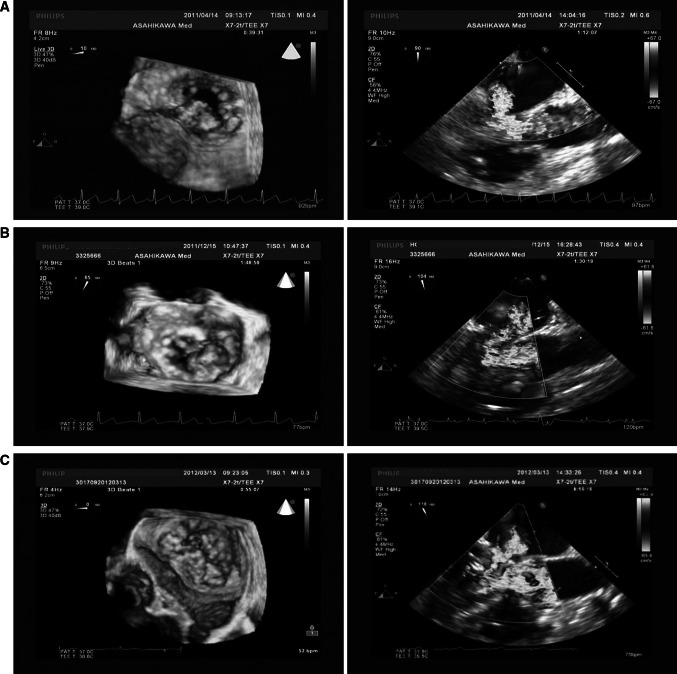
**a** Preoperative 3D-TEE and 2D-TEE mid-esophageal long-axis view at the appearance of SAM in case 1. 3D-TEE indicated a vegetation and prolapse of posterior leaflet. **b** Preoperative 3D-TEE and 2D-TEE mid-esophageal long-axis view at the appearance of SAM in case 2. 3D-TEE indicated prolapse of posterior leaflet, P2. **c** Preoperative 3D-TEE and 2D-TEE mid-esophageal long-axis view at the appearance of SAM in case 3. 3D-TEE indicated prolapse of posterior leaflet

A 65-year-old woman had fever and visual deficit, and thorough testing confirmed the presence of endocarditis, including MR and cerebral infarction. Preoperative echocardiography confirmed moderate MR due to prolapse of the posterior leaflet and vegetation (9 × 4 mm) (Fig. 1a). The ejection fraction was 65 %; thus, MVP was indicated.

Anesthesia was induced with target controlled infusion (TCI) of 3 μg/ml propofol, 0.3 μg/kg/min remifentanil, and 40 mg rocuronium, with timely administration of phenylephrine. Preoperative transesophageal echocardiography (TEE) was performed for SAM (Table 1). The distance from the septum to the mitral valve coaptation point (C-sept) was 2.2 cm. Low dose landiolol (3 μg/kg/min) was administered at the start of surgery. Quadrangular resection, suturing of the posterior mitral valve leaflet (PML), and vegetation resection were performed. The patient was weaned from cardiopulmonary bypass (CPB) with 5 μg/kg/min dopamine, and 5 μg/kg/min dobutamine.

**Table 1:** Preoperative morphological risk factor of SAM

Risk factor	Case 1	Case 2	Case 3
AL/PL ratio <1.3	1.3	1.1*	0.8*
PML >15 mm	10 mm	16 mm*	27 mm*
AML >27 mm	13 mm	18 mm	23 mm
Distance between coaptation and interventricular septum (C-sept) <25 mm	22 mm*	23 mm*	18 mm*
Small angle between the aortic and mitral annular planes (<120°)	143°	123°	124°
Small ventricle (<36 mm)	44 mm	48 mm	45 mm
Thickening of interventricular septum	(–)	(–)	(–)
Small angle between AML and outflow	27°	25°	20°
Small distance between coaptation and outflow	10 mm	8 mm	5 mm

Asterisk indicates the risk factor of SAM

After separation from CPB, blood pressure suddenly became unstable at 74/34 mmHg. TEE indicated SAM (Fig. 1a). We stopped the administration of catecholamines and starting noradrenaline administration. A bolus of 6 mg landiolol was initiated at 10 μg/kg/min. TEE confirmed the disappearance of SAM, and hemodynamics improved. Upon disappearance of SAM, the predicted plasma landiolol concentration was 0.28 μg/ml according to the Stanpump software.

## Case 2

A 53-year-old woman had no complaint, but exhibited cardiac murmur; thorough testing confirmed MR. Severe MR due to prolapse of the posterior leaflet was confirmed by preoperative echocardiography (Fig. 1b). The ejection fraction was 67 %; thus, MVP was indicated.

Anesthesia was induced with TCI of 3 μg/ml propofol, 0.3 μg/kg/min remifentanil, and 40 mg rocuronium, with timely administration of phenylephrine. Preoperative TEE was performed in considering SAM (Table 1). The C-sept was 2.3 cm. Triangular resection and suturing of the PML were performed. The patient was weaned from CPB with 3 μg/kg/min dopamine and 3 μg/kg/min dobutamine.

After separation from CPB, her blood pressure suddenly became unstable at 60/40 mmHg. TEE indicated SAM (Fig. 1b). We decreased the dose of catecholamines, and injected two boluses of 5 mg landiolol at 10 μg/kg/min. Then SAM continued, so the landiolol dose was increased to 20 μg/kg/min. TEE confirmed the disappearance of SAM. Upon the disappearance of SAM, the predicted plasma landiolol concentration was 0.40 μg/ml, according to the Stanpump software.

## Case 3

A 55-year-old man had no complaint, but exhibited cardiac murmur; thorough testing confirmed MR. Preoperative echocardiography was confirmed to be severe MR due to prolapse of the posterior leaflet and rupture of the chordae tendineae (Fig. 1c). The ejection fraction was 62 %; thus, MVP was indicated.

Anesthesia was induced at 4 μg/ml TCI of propofol, 0.4 μg/kg/min remifentanil, and 50 mg rocuronium, with timely administration of phenylephrine. Preoperative TEE was performed considering SAM (Table 1). The C-sept was 1.8 cm, the AL/PL ratio was 0.8, and the length of PML was 27 mm. A quadrangular resection and suturing of the PML was performed. The patient was weaned from CPB with 2 μg/kg/min dopamine and 4 μg/kg/min dobutamine.

**Fig. 2:**
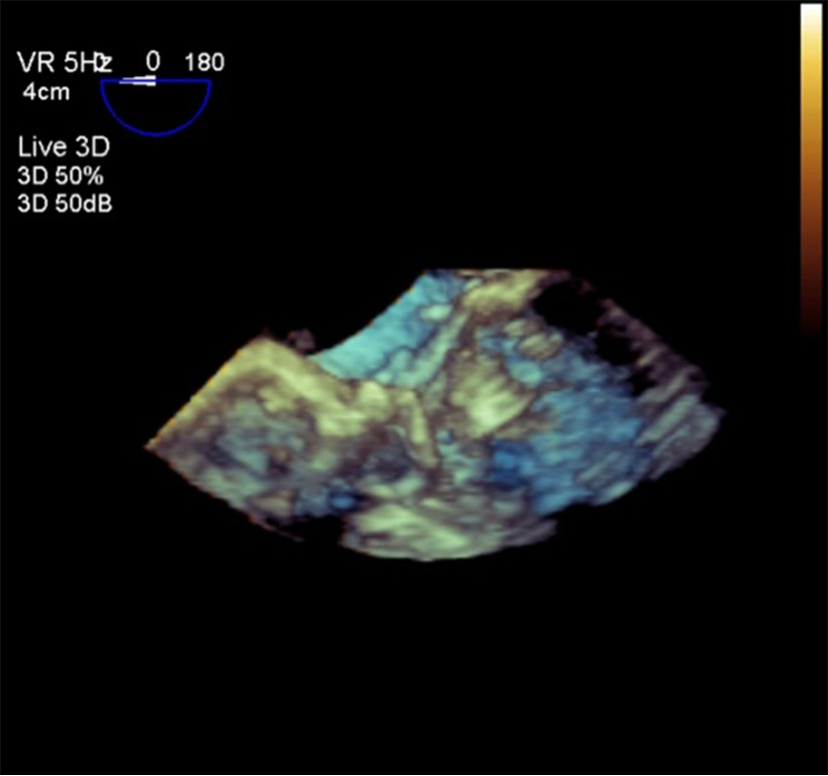
It showed real time 3D-TEE when SAM is occurring in case 3

After separation from CPB, blood pressure suddenly became unstable at 70/40 mmHg. TEE indicated SAM (Fig. 1c). It showed real time 3D-TEE when SAM was occurring (Fig. 2). We decreased the catecholamine dose, started noradrenaline and administered two boluses of 3 mg landiolol at 7 μg/kg/min. TEE confirmed the disappearance of SAM, and hemodynamics improved. Upon the disappearance of SAM, the predicted plasma landiolol concentration was 0.22 μg/ml according to the Stanpump software.

## Discussion

The mechanism of SAM has been reported as a Venturi or drag effect by Cape et al. and Sherrid et al. [10–12]. But its precise mechanism has not been elucidated.

The appearance of SAM is a major complication following MVP and is reported to occur in from 5 to 10 % of cases [13]. The morphological characteristics of SAM have been revealed by several studies. Preoperative mitral valve morphology is a risk factor for SAM. Maslow et al. [3] indicated that the pre-repair AL/PL ratio in SAM cases was <1.3 and C-sept was <2.5 cm. Mihaileanu et al. [14] reported that the angle between the aortic and mitral annular planes was <120°in SAM cases. Orihashi [5] indicated the distance between the outflow and coaptation was small and that the angle between the anterior mitral leaflet (AML) and outflow also was small in SAM cases. Quigloey et al. [15] reported that the AML was >27 mm, and that the PML should be 15 mm in SAM cases. In our cases, measurements were performed in all three cases of SAM (Table 1). As a result, the C-sept was <2.5 cm in all three cases. However, these morphology related reports are based on upper and lower limits, means, and experiences, without clearly setting cut-off values; it may be necessary to consider these values as references, rather than absolute standards, and combine them for the prediction of onset of SAM.

TEE was useful for distinguishing SAM from residual MR. The appearance and disappearance of SAM were certainly identified by TEE (Fig. 1a, b, c). Recently, 3D-TEE has been useful and provides additional information [16]. Figure 2 shows real time 3D-TEE when SAM is occurring in Case 3. With the use of 3D-TEE, when mitral valve prolapse was investigated, in all three cases, it was easy to specify lesions. The issue for the future is 3D analysis when SAM is occurring.

Treatment for SAM increases preload and afterload, specifically transfusion, and using an α-adrenergic agent decreases heart rate, while stopping dopamine and dobutamine. However, transfusions, starting a continuous infusion of noradrenaline, and limiting dopamine and dobutamine were insufficient in all three of our cases.

Landiolol is a cardioselective ultra short-acting β1-adrenergic receptor blocking agent. This drug is hydrolyzed to an inactive form by both carboxyesterase in the liver and pseudocholinesterase in plasma. It has a shorter plasma half-life of approximately 4 min compared with that of esmolol (9 min). Landiolol has much higher cardioselectivity (β1/β2 = 255) than that of esmolol (β1/β2 = 33) [17]. When patients are weaned from CPB, drugs that strictly control these effects are more desirable. Landiolol concentrations rapidly reached steady state levels and then rapidly dissipated after completing administration.

It is possible to predict plasma landiolol concentrations at the disappearance of SAM. The predicted plasma landiolol concentrations in cases 1–3 were 0.28, 0.4, and 0.22 μg/ml, respectively.

The cause of improvement in SAM is not only landiolol; a variety of factors have influence. We would be pleased, however, if the concentrations in our paper could serve as reference values into the future.
